# Determination and Daily Intake Estimation of Lignans in Sesame Seeds and Sesame Oil Products in Korea

**DOI:** 10.3390/foods9040394

**Published:** 2020-03-30

**Authors:** A-Young Kim, Choong-In Yun, Joon-Goo Lee, Young-Jun Kim

**Affiliations:** 1 Department of Food Science and Technology, Seoul National University of Science and Technology, 232 Gongneung-ro, Nowon-gu, Seoul 01811, Korea; ayung0814@seoultech.ac.kr; 2 Lab of Nanobio, Seoul National University of Science and Technology, 232 Gongneung-ro, Nowon-gu, Seoul 01811, Korea; innny@naver.com; 3 Food Standard Division, Ministry of Food and Drug Safety, Cheongju 28159, Korea

**Keywords:** sesamol, sesaminol, sesamin, sesamolin, lignin intake

## Abstract

Sesame (*Sesamum indicum* L.) is a plant that belongs to the *Pedaliaceae* family which was first classified as a food source around 4000 years ago. Lignans (sesamin, sesamolin, sesamol, and sesaminol) present in sesame are the primary functional compounds that impart important health benefits. However, very little information is available on the lignan intake from sesame seeds and sesame oil products. Sesame oil is frequently and highly consumed in Korea and therefore is one of the important lignan intake sources due to the food eating habits of Koreans. Herein, we studied the distribution of lignans in sesame seeds (*n* = 21) and oil (*n* = 34) to estimate the daily lignan intake by the Korean population. High-performance liquid chromatography, in conjunction with statistical analysis, was used to determine the lignan content of seeds and oil. The estimated daily intake of total lignans from sesame seeds and oil, as estimated from the available domestic consumption data (Korea Nutrition and Health Examination Survey), is 18.39 mg/person/day for males and 13.26 mg/person/day for females. The contributions of lignan intake from sesame seeds and oil are 23.0% and 77.0%, respectively. This study provides preliminary information on lignan intake from sesame seeds and oil in the Korean population.

## 1. Introduction

Sesame (*Sesamum indicum* L.) is a plant from the *Pedaliaceae* family, which was first classified as a food source from Assyria and Babylon around 4000 years ago. It is one of the oldest cultivated food crops by humans [1]. In 2011, sesame production amounted to 4.092 million tons, while the total harvested area of sesame plants in the world was 6.628 million ha [2]. The major sesame production regions are the Asian and African countries, such as India, China, Myanmar, Sudan, and Ethiopia [1]. Approximately 35% of the annual production of sesame is consumed as a food ingredient, while 65% is used as an edible oil for food preparation [1]. As reported by Korean trade statistics for the period from 2014 to 2016 [3], sesame seeds were imported majorly from India (46.1%), followed by China (27.8%), Ethiopia (13.3%), Nigeria (3.6%), Pakistan (3.1%), Burkina Faso (2.2%), Sudan (0.8%), and Paraguay (0.8%). In 2016, a total of 77.906 metric tons (equivalent to 111.1 million USD) of sesame seeds were imported, which was 1.1 times more than that imported in 2000 (70.118 tons). The import, in 2000, corresponded to a monetary value of USD 52.5 million, which represented an increase of 2.1 times over 16 years. The reason for the increase in sesame seeds import in 2016 is its unique and distinct flavor that is very popular in Korean cuisines.

Interest in dietary lignans has been increasing due to their potential beneficial properties. Lignans have been identified in a variety of foods such as vegetables, spices, herbs, fruits, etc. [4]. Several bioactive compounds have been identified in sesame such as phenolics, phytosterols, phytates, lignans, and tocopherol [5]. Especially, the lignans (sesamin, sesamolin, sesamol, and sesaminol) present in sesame seed are primary functional compounds that have properties which are beneficial to human health [6]. Sesame lignans have been identified as having antioxidants, antimicrobial preservatives, anti-inflammatory, and anticancer properties [7,8,9]. The potent antioxidant capacity of lignans such as sesamol, sesamin, and sesamolin can reduce the oxidation of oil and methyl linoleate at relatively low temperatures [10,11,12]. Sesamin and sesamolin are the main soluble lignans, and the oxidative stability of the oil and seeds is mostly due to these components. The ability of these lignans to prevent the rancidity of sesame oil extends the shelf life of the products [13]. Moreover, sesame oil can be used in cosmetics due to its bioactivity such as tyrosinase, elastase, collagenase, and hyaluronidase inhibition activity [14]. According to Moazzami and Kamal-Eldin [15], sesamin, sesamolin, and sesaminol account for 0.26% to 1.16% of the lignans in the sesame seeds. A minor component of the lignans is sesamol [16].

Currently, there are numerous methods for the quantification analysis of individual lignans, including high-performance liquid chromatography/ultraviolet spectroscopy [2,6,13,16,17,18,19,20,21], liquid chromatography-mass spectrometry [17], liquid chromatography-electrospray ionization tandem mass spectrometry [22], and non-destructive analysis using near-infrared analysis [23]. However, there is very little data on the estimation of lignan intake from sesame seeds and sesame oil products. The dietary intake amounts of compounds via food consumption are more important than their concentrations in food. Sesame oil is frequently and highly consumed in Korea. Especially, sesame seeds and sesame oil contain unique lignans such as sesamin, sesamolin, sesamol, and sesaminol. Although there are some foods that contain lignans such as flaxseed, sesame oil is one of the important lignan intake sources due to the food eating habits of Koreans. Therefore, there is a need to estimate the dietary intake of lignans from sesame seeds and sesame oil.

The objectives of this study are (1) to improve and validate the simultaneous analytical determination of individual lignans using high-performance liquid chromatography with a diode-array detector (HPLC-DAD), (2) to apply the suggested method for the quantification of sesame seeds and sesame oil products, and (3) to assess the estimated daily intake (EDI) of lignans from sesame seeds and sesame oil products.

## 2. Materials and Methods

### 2.1. Reagents and Chemicals

The solvents used were of analytical high-performance liquid chromatography (HPLC) grade. Methanol, chloroform, and *n*-hexane were purchased from Fisher Scientific (Pittsburgh, PA, USA). A Milli-Q water system (Millipore, MA, USA) was used to purify the deionized water (18.2 MΩ) for the HPLC mobile phase. Before analysis, the HPLC solvents were filtered through 47 mm, 0.45 μm HVLP filters (Millipore, MA, USA), and the extracted samples were filtered through 15 mm, 0.45 μm regenerated cellulose (RC) membrane filters (Sartorius, Göttingen, Germany). The lignan reference standards (sesamin, sesamolin, sesamol, sesaminol) were purchased from Nagara Science Co. (Furuichiba, Gifu, Japan).

### 2.2. Sample Collection

Sesame seeds and sesame oil used in this study were the predominant sources of lignans [24]. Commercial grade sesame seeds (total *n* = 21), harvested in India (*n* = 7), China (*n* = 3), Korea (*n* = 3), Ethiopia (*n* = 2), Pakistan (*n* = 2), Sudan (*n* = 2), Nigeria (*n* = 1), and Paraguay (*n* = 1) from 2015 to 2017 were provided by The Ottogi Sesame Mills Co., Ltd. (Choongnam, Korea). Each sesame seed sample of 500 g was powdered using a blender (HR 2860, Philips, Shanghai, China) for sample preparation. The powdered sesame seeds were stored at 4 °C until analysis. Additionally, thirty-four sesame oil products were obtained from retail markets in Anyang City, Korea, from 2015 to 2017, and these oil samples were also stored at 4 °C until analysis.

### 2.3. Sample Preparation for Lignans 

The powdered sesame seeds (5.0 g) were added to 50 mL *n*-hexane using an extraction cellulose thimble. Then, the sample was boiled for 90 min at 135 °C by the Soxhlet method using a FOSS Soxtec 2050 (Foss Tecator, Höganäs, Sweden). After extraction of samples, the samples were stored at 25 °C in a desiccator; then, the extracted oil was weighed for lignan analysis [22]. The oil content was measured and expressed as g/100 g. Commercial grade sesame seeds oil or the crude sesame extract oil from the Soxhlet method (about 1 g) was dissolved in 10 mL chloroform in a 20 mL volumetric flask. Then, the volumetric flask was filled with methanol, and the solution was mixed by a vortex mixer. Following this, the sample solution was diluted 10-fold with methanol and filtered through a 0.45 μm syringe filter before HPLC analysis.

### 2.4. HPLC Analysis of Individual Lignans

HPLC using C18 columns has previously been used for the separation of individual lignans in sesame seeds and oil [11,25]. However, due to similar chemical characteristics, it is difficult to separate the individual lignans. Here, the lignans were analyzed using methods described previously, with modifications [6,21,26]. The individual lignans were analyzed on a high-performance liquid chromatograph (Agilent 1100 series, Palo Alto, CA, USA) equipped with a degasser, quaternary pump, autosampler, column oven, and diode array detector. ZORBAX Eclipse Plus C18 (5 μm, 150 × 4.6 mm i.d.; Agilent Technology, Santa Clara, CA, USA), a reversed-phase column, was used. The HPLC mobile phase consisted of mobile phase A (water) and B (methanol), and the samples were eluted using the following gradient: 0 min, 30% B; 0−4 min, 3% to 80% B; 5−10 min, 18% to 35% B; 10−15 min, 35% to 62% B; 15−18 min, 62% to 80% B, 18−22 min, 80% B; and 22−23 min, 80% to 5% B. The column was equilibrated with 30% mobile phase B for 5 min at 25 °C. The flow rate, injection volume, and detection wavelength were 1.0 mL/min, 20 μL, and 290 nm, respectively. The retention time and peak area of each standard lignan compound were used for the quantification and qualification of the results. 

### 2.5. HPLC Method for Validation

The HPLC method for validation was conducted to confirm calibration parameters (range, slope, intercept, linearity, and the limit of detection (LOD), the limit of quantification (LOQ)), precision (intraday and interday), and accuracy (recovery test). Linearity analysis was performed in the ranges 0.3−25 and 0.3−50 mg/kg for sesamol and sesaminol, sesamin and sesamolin, respectively. A signal to noise (S/N) ratio was used for the LOD (S/N, 3) and LOQ (S/N, 10), respectively. Precision (intraday for repeatability and interday for reproducibility) was determined by analyzing the commercial sesame oil sample six times per day and three times on three different days for reproducibility tests. Then, it was expressed as the percent relative standard deviation (% RSD) for precision. The accuracy (recovery test) was determined by following the entire procedure using fortified samples which contained sesamol, sesaminol, sesamin, and sesamolin at 100, 100, 2500, and 4000 mg/kg.

### 2.6. Estimation of Daily Intake of Lignans

Sesame seeds and sesame oil are a primary dietary source of lignan intake [21]. The EDI of lignan intake from sesame seeds, Equation (1); the EDI of lignan intake from sesame oil, Equation (2); and the total EDI of lignans from all sesame products, Equation (3) were calculated as follows:

EDI of lignans from sesame seeds (mg/person/day) = average content of lignans in sesame seeds (mg/g) × consumption of sesame seeds (g) per day(1)

EDI of lignans from sesame oil (mg/person/day) = average content of lignans in sesame oil (mg/g) × consumption of sesame oil (g) per day(2)

Total EDI of lignans from sesame products (mg/person/day) = EDI of lignans from sesame seeds (mg/person/day) + EDI of lignans from sesame oil (mg/person/day)(3)

The consumption of sesame seeds and sesame oil data was obtained from the Korea National Health and Nutrition Examination Survey (KNHANES, Cheongju, Korea) in 2016 from both genders in 3513 Korean households [27]. The consumption data were primarily categorized into male and female, which were further categorized into age groups 1−2, 3−5, 6−11, 12−18, 19−29, 30−49, 50−64, and over 65 years old (*n* = 7049).

### 2.7. Statistical Analysis. 

All the data were expressed as the mean ± standard deviations (SD) of triplicate analyses. Analysis of variance was performed using SAS version 9.3 (SAS Institute, Inc., Cary, NC, USA) for the statistical analysis of each experiment. Duncan’s multiple range test determined the significance of differences (*p* < 0.05). The correlation was determined using Pearson’s correlation coefficient.

## 3. Results and Discussion

### 3.1. HPLC Method for Validation

The HPLC method for validation was performed before data collection to ensure the separation and quantitative analysis of the individual lignans. The retention times for sesamol, sesaminol, sesamin, and sesamolin were 6.75, 29.85, 33.30, and 36.21 min, respectively. To overcome the difficulties in chromatographic separation and achieve efficient resolution, the samples were eluted using a mobile phase gradient. The calibration range, linear regression equations of slope and intercept, correlation coefficients (*r^2^*), LOD, and LOQ are shown in Table 1. The LOD and LOQ were in the range 0.08−0.11 mg/kg and 0.26−0.36 mg/kg, respectively.

The recovery test results for sesame oil samples are listed in Table 2. The recovery results ranged from 94.38% to 102.94% (average 97.92%), which complies with the range permitted by Food and Drug Administration (FDA) guidelines (80% to 120%) [28].

Precision results were obtained using repetitive measurements. Table 3 shows that the relative standard deviation (RSD) of six replicate intraday experiments ranged between 0.29% and 0.53% (repeatability). The nine replicated interday experiments (conducted for 3 days) also showed less than the maximum of 0.85% RSD (reproducibility). The percentage RSD was calculated by dividing the SD by the average value, and then multiplying by 100.

### 3.2. Lignan Contents in Sesame Seeds and Oil

**Figure 1:**
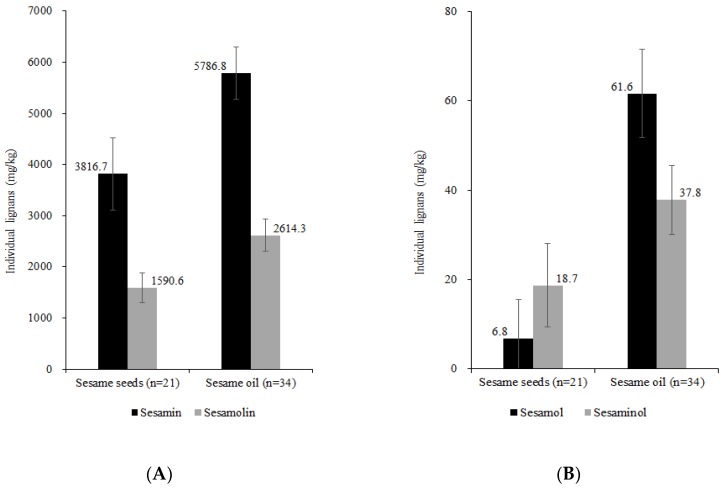
Individual lignan contents in sesame seeds (*n* = 21) and sesame oil (*n* = 34). (**A**) Sesamin and sesamolin; (**B**) Sesamol and sesaminol.

The results of the HPLC analysis of sesame seed samples (*n* = 21) and sesame oil samples (*n* = 34) are shown in Figure 1. Figure 1A shows the sesamin and sesamolin contents, while Figure 1B shows the sesamol and sesaminol contents in sesame seeds and sesame oil.

In sesame seeds, the sesamin, sesamolin, sesamol, and sesaminol contents ranged from 1568.4 to 7746.8 mg/kg (average 3816.7 ± 1414.2 mg/kg), from 720.1 to 3176.1 mg/kg (average 1590.6 ± 581.3 mg/kg), from 0 to 70.3 mg/kg (average 6.8 ± 17.2 mg/kg), and from 0 to 63.9 mg/kg (average 18.7 ± 18.7 mg/kg), respectively. The total lignans in sesame seeds ranged from 2306.2 to 10,973.5 mg/kg with an average content of 5432.9 ± 1963.3 mg/kg. Previous studies on sesame seeds from different countries have shown a varied range for each lignan. In the sesame seeds (*n* = 3) from Taiwan, 2.16 ± 0.10 and 1.38 ± 0.10 mg/g sesamin and sesamolin were detected, respectively [26]. The average contents (*n* = 3) of sesamol, sesamin, and sesamolin in sesame seeds from New Delhi, India, were 78.34 ± 77.69, 279.88 ± 100, and 129.81 ± 43.10 mg/100 g, respectively [13]. The sesame seeds (*n* = 13) from China had sesamin and sesamolin contents in the ranges 2.38−9.32 mg/g (average 6.34 mg/g) and 2.47−5.27 mg/g (average 3.53 mg/g), respectively [19].

In sesame oil, the sesamin and sesamolin contents ranged from 4112.3 to 7523.2 mg/kg (average 5786.8 ± 1029.3 mg/kg) and from 1818.0 to 4071.5 mg/kg (average 2614.3 ± 633.8 mg/kg), respectively. The sesamol and sesaminol contents were in the ranges 9.8−108.7 mg/kg (average 61.6 ± 19.8 mg/kg) and 8.8−62.7 mg/kg (average 37.8 ± 15.4 mg/kg). The total lignans in sesame oil ranged from 6059.9 to 11479.9 mg/kg, with an average content of 8500.5 ± 1564.7 mg/kg. In previous studies on sesame oil, the sesamin, sesamolin, and sesamol contents were found to be 3,340 ± 14, 1,964 ± 11, and 452 ± 4 mg/kg, respectively, and the total lignans were 5756 ± 29 mg/kg [16]. Jeon et al. [18] reported that the total lignan contents in Korean (*n* = 51), Chinese (*n* = 19), and Indian (*n* = 14) sesame oil ranged from 363.0 to 626.9 mg/100 g (average 467.6 mg/100 g), from 232.2 to 324.6 mg/100 g (average 275.4 mg/100 g), and from 282.0 to 519.7 mg/100 g (average 436.6 mg/100 g), respectively. In the case of Taiwanese sesame oil, the average sesamin and sesamolin contents were 9.47 ± 2.28 and 1.74 ± 0.76 mg/g, respectively [6]. Moreover, sesamin, sesamolin, and total lignan contents in Indian sesame (*n* = 40) ranged from 0.08 to 2.58 mg/g, 0.28 to 2.52, and 0.49 to 4.55 mg/g, respectively [29].

These results showed that lignan content varies over a wide range, which is similar to the results obtained in this study. Among the sesame seeds and oil samples, the relative percentage of each lignan was as follows: sesamin 68.6%, sesamolin 30.4%, sesamol 0.6%, and sesaminol 0.4%.

### 3.3. Correlation

**Figure 2:**
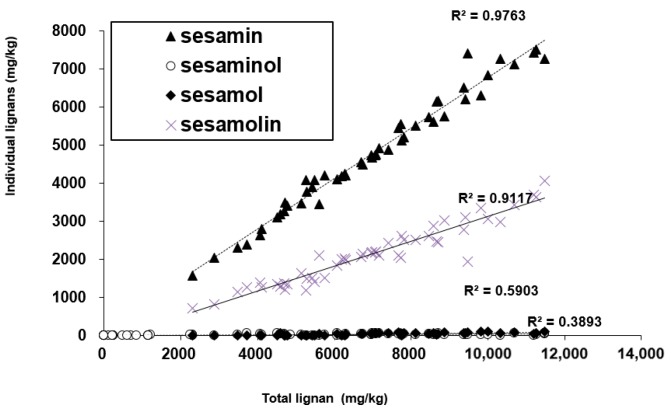
Correlation between total lignans and individual lignans in sesame seeds and sesame oil.

A previous study has been conducted to obtain a correlation between the lignan contents in sesame seeds [15]. In this study, Pearson correlations were calculated for both sesame seeds (*n* = 21) and sesame oil (*n* = 34) to determine the relationship between sesamin and sesamolin contents (Table 4). The correlations were determined to be the highest between sesamin and sesamolin, with R^2^ = 0.914 in sesame seeds and R^2^ = 0.765 in sesame oil. The correlations obtained in our study were a little higher than those in the previous study (R^2^ values of 0.66 in white sesame seeds and 0.77 in black sesame seeds) by Moazzami and Kamal-Eldin [15]. Additionally, Figure 2 shows that sesamin and sesamolin have a strong correlation between the total lignan content in the sesame seed and oil. Particularly, the levels of the total lignan content strongly depend on the content of sesamin. Therefore, sesamin can be the primary indicator of the quality of sesame seeds and oil.

### 3.4. Assessment of the Estimated Daily Intake of Lignans 

According to KNHANES, in 2016 the average daily intake of sesame seeds was 0.71 and 0.61 g/person/day for males and females, respectively [27]. Moreover, the average daily intake of sesame oil was 1.71 and 1.17 g/person/day for males and females, respectively. The EDI of lignans by individuals was evaluated by multiplying the consumption by the average content of each lignan in the sesame seeds and oil. The average daily consumption of sesame seeds and sesame oil and their corresponding EDI for each age group are available in Table 5 and Table 6. In sesame seeds, the EDI values for males and females (estimated daily intake) are 2.710 and 2.328 mg/person/day for sesamin, 1.129 and 0.970 mg/person/day for sesamolin, 0.005 and 0.004 mg/person/day for sesamol, and 0.013 and 0.011 mg/person/day for sesaminol, respectively. Therefore, the EDI of lignans from sesame seeds was 3.86 mg/person/day for males and 3.31 mg/person/day for females (Table 5).

In sesame oil, the EDI values for males and females are 9.895 and 6.771 mg/person/day for sesamin, 4.470 and 3.059 mg/person/day for sesamolin, 0.105 and 0.072 mg/person/day for sesamol, and 0.065 and 0.044 mg/person/day for sesaminol, respectively. Therefore, EDI of lignans from sesame oil was 14.54 mg/person/day for males and 9.95 mg/person/day for females (Table 6).

For calculating the total EDI of lignans in sesame seeds and sesame oil, the sum of sesame seeds and sesame oil intake was used. The total EDI values for males and females from sesame seeds and sesame oil are 12.605 and 9.099 mg/person/day for sesamin, 5.599 and 4.029 mg/person/day for sesamolin, 0.110 and 0.076 mg/person/day for sesamol, and 0.078 and 0.055 mg/person/day for sesaminol, respectively. Therefore, it was concluded that the total EDI average values of lignans from sesame seeds and sesame oil was 18.39 mg/person/day for males and 13.26 mg/person/day for females. Comparatively, the males average EDI was 1.4 times higher than that of the females (Table 7). There was an increasing trend by age, with the highest intake of 21.756 mg/person/day between the ages of 30 and 49 years, for males. In addition, the lignan intake had decreased at the age of 50. In the same way, females also showed a similar tendency to males, with the highest intake of 16.483 mg/person/day between 30 and 49 years old. After 50 years old, the tendency had dwindled. These trends could be attributed to the high intake of sesame seeds and sesame oil in the Korean dietary eating pattern. These preliminary evaluation results of EDI, based on an HPLC analysis approach and assessment of EDI, represent necessary information on lignan intake from sesame seeds and sesame oil in the Korean diet.

Table 8 shows the average contribution (in percentage) of lignans in sesame seeds and sesame oil. Males consumed 14.7% and 53.8% sesamin from sesame seeds and sesame oil, respectively, and 6.1% and 24.3% sesamolin in sesame seeds and sesame oil, respectively. Females consumed 17.6% and 51.1% sesamin from sesame seeds and sesame oil, respectively, and 7.3% and 23.1% sesamolin from sesame seeds and sesame oil, respectively. The total average composition of sesamin was 16.1% and 52.4% from sesame seeds and sesame oil, respectively, while that of sesamolin was 6.7% and 23.7% from sesame seeds and sesame oil, respectively. The contribution of lignan intake from both sesame seeds and oil were 23.0% and 77.0% of the total, respectively. The assessment of the data reveals that the dominant source of lignans for intake by people is the sesame oil.

## 4. Conclusions

This study presents a method for the simultaneous determination and quantification of four bioactive lignans in sesame oil and seeds. Reversed-phase HPLC-DAD with gradient elution is a reliable analytical method and was used for the analysis of lignans in samples. The total lignan contents in different samples varied over a wide range because of different origins and harvesting conditions. It was determined that sesamin could be the primary indicator of the quality of sesame seeds and oil with a strong correlation with total lignans. Additionally, this study presents a preliminary assessment of the EDI of unique lignans including sesamin, sesamolin, sesamol, and sesaminol from sesame oil and seeds in the Korean diet. The total EDI average values of lignans from sesame seeds and sesame oil is 18.39 mg/person/day for males and 13.26 mg/person/day for females. Comparatively, the males’ average EDI was 1.4 times higher than that of the females. The ages between 30 and 49 years old showed the highest lignan intake.

## Figures and Tables

**Table 1:** Calibration parameters obtained for individual lignans.

Compounds	Range (mg/kg)	Slope	Intercept	Correlation Coefficient (r^2^)	LOD ^1^ (mg/kg)	LOQ ^2^ (mg/kg)
Sesamol	0.3−25	30.55	1.08	0.9999	0.10	0.33
Sesaminol	0.3−25	25.35	0.47	0.9999	0.08	0.26
Sesamin	0.3−50	24.21	0.57	0.9999	0.09	0.31
Sesamolin	0.3−50	27.05	0.97	0.9999	0.11	0.36

^1^ LOD, limit of detection; ^2^ LOQ, limit of quantification.

**Table 2:** Accuracy results for individual lignans spiked in sesame oil.

Compounds	Present (mg/kg)	Added (mg/kg)	Found (mg/kg)	Recovery (%)	RSD (%)
Sesamol	59.4	100	154.94 ± 0.29	95.56 ± 0.29	0.31
Sesaminol	62.5	100	156.92 ± 0.04	94.38 ± 0.04	0.04
Sesamin	5250.1	2500	7823.63 ± 2.42	102.94 ± 3.15	3.06
Sesamolin	1893.9	4000	5845.44 ± 12.09	98.79 ± 2.01	2.04

**Table 3:** Precision of the intraday and interday high-performance liquid chromatography (HPLC) measurement for individual lignans.

Compounds	Intraday		Interday	
	Contents (mg/kg)	RSD (%)	Contents (mg/kg)	RSD (%)
Sesamol	59.15 ± 0.17	0.29	59.38 ± 0.50	0.85
Sesaminol	62.53 ± 0.20	0.31	62.54 ± 0.20	0.31
Sesamin	5272.15 ± 27.69	0.53	5250.07 ± 27.52	0.52
Sesamolin	1893.59 ± 3.30	0.40	1893.89 ± 4.24	0.22

**Table 4:** Pearson correlation between compounds of sesame seeds and oil.

Type	Compounds	Sesaminol	Sesamin	Sesamolin	Total
Seeds	Sesamol	0.071 ^ns^	0.544 ^*^	0.486 ^*^	0.545 ^*^
	Sesaminol	-	−0.358 ^ns^	−0.215 ^ns^	−0.311 ^ns^
	Sesamin	-	-	0.914^**^	0.992 ^**^
	Sesamolin	-	-	-	0.957 ^**^
Oil	Sesamol	0.122 ^ns^	0.268 ^ns^	0.252 ^ns^	0.292 ^ns^
	Sesaminol	-	−0.564 ^**^	−0.534 ^**^	−0.576 ^**^
	Sesamin	-	-	0.765 ^**^	0.965 ^**^
	Sesamolin	-	-	-	0.906 ^**^

Pearson correlation significance at ** *p* < 0.01 and * *p* < 0.05; ns, no significance.

**Table 5:** Estimated daily intake (EDI) of lignans from sesame seeds (mg/person/day).

Gender	Ages	Consumption ^1^ (g/day)	Estimated Daily Intake of Lignans ^2^				
			Sesamin	Sesamolin	Sesamol	Sesaminol	Total
Males	1−2	0.24	0.916	0.382	0.002	0.004	1.304
	3−5	0.43	1.641	0.684	0.003	0.008	2.336
	6−11	0.72	2.748	1.145	0.005	0.013	3.912
	12−18	0.88	3.359	1.400	0.006	0.016	4.781
	19−29	0.81	3.092	1.288	0.006	0.015	4.401
	30−49	0.75	2.863	1.193	0.005	0.014	4.075
	50−64	0.69	2.634	1.098	0.005	0.013	3.749
	>65	0.53	2.023	0.843	0.004	0.010	2.879
	Average	0.71	2.710	1.129	0.005	0.013	3.857
Females	1−2	0.19	0.725	0.302	0.001	0.004	1.032
	3−5	0.3	1.145	0.477	0.002	0.006	1.630
	6−11	0.53	2.023	0.843	0.004	0.010	2.879
	12−18	0.77	2.939	1.225	0.005	0.014	4.183
	19−29	0.40	1.527	0.636	0.003	0.007	2.173
	30−49	0.64	2.443	1.018	0.004	0.012	3.477
	50−64	0.74	2.824	1.177	0.005	0.014	4.020
	>65	0.60	2.290	0.954	0.004	0.011	3.260
	Average	0.61	2.328	0.970	0.004	0.011	3.314

^1^ 2016 report on Korea National Health and Nutrition Examination Survey. ^2^ Average content of individual lignans in sesame seeds (mg/g) x consumption of sesame seed (g) a day.

**Table 6:** Estimated daily intake (EDI) of lignans from sesame oil (mg/person/day).

Gender	Ages	Consumption ^1^ (g/day)	Estimated Daily Intake of Lignans ^2^				
			Sesamin	Sesamolin	Sesamol	Sesaminol	Total
Males	1−2	0.82	4.745	2.144	0.051	0.031	6.970
	3−5	1.04	6.018	2.719	0.064	0.039	8.841
	6−11	1.11	6.423	2.902	0.068	0.042	9.436
	12−18	1.36	7.870	3.555	0.084	0.051	11.561
	19−29	1.79	10.358	4.680	0.110	0.068	15.216
	30−49	2.08	12.037	5.438	0.128	0.079	17.681
	50−64	1.84	10.648	4.810	0.113	0.070	15.641
	>65	1.16	6.713	3.033	0.071	0.044	9.861
	Average	1.71	9.895	4.470	0.105	0.065	14.536
Females	1−2	0.63	3.646	1.647	0.039	0.024	5.355
	3−5	0.89	5.150	2.327	0.055	0.034	7.565
	6−11	0.94	5.440	2.457	0.058	0.036	7.990
	12−18	1.06	6.134	2.771	0.065	0.040	9.011
	19−29	0.99	5.729	2.588	0.061	0.037	8.416
	30−49	1.53	8.854	4.000	0.094	0.058	13.006
	50−64	1.17	6.771	3.059	0.072	0.044	9.946
	>65	0.83	4.803	2.170	0.051	0.031	7.055
	Average	1.17	6.771	3.059	0.072	0.044	9.946

^1^ 2016 report on Korea National Health and Nutrition Examination Survey ^2^ Average content of individual lignans in sesame oil (mg/g) x consumption of sesame oil (g) a day.

**Table 7:** Total estimated daily intake (EDI) of lignans from sesame seeds and sesame oil (mg/person/day).

Gender	Ages	Estimated Daily Intake of Lignans ^1^				
		Sesamin	Sesamolin	Sesamol	Sesaminol	Total
Males	1−2	5.661	2.526	0.053	0.035	8.274
	3−5	7.659	3.403	0.067	0.047	11.177
	6−11	9.171	4.047	0.073	0.055	13.348
	12−18	11.229	4.955	0.090	0.067	16.342
	19−29	13.45	5.968	0.116	0.083	19.617
	30−49	14.9	6.631	0.133	0.093	21.756
	50−64	13.282	5.908	0.118	0.083	19.390
	>65	8.736	3.876	0.075	0.054	12.740
	Average	12.605	5.599	0.110	0.078	18.393
Females	1−2	4.371	1.949	0.040	0.028	6.387
	3−5	6.295	2.804	0.057	0.040	9.195
	6−11	7.463	3.300	0.062	0.046	10.869
	12−18	9.073	3.996	0.070	0.054	13.194
	19−29	7.256	3.224	0.064	0.044	10.589
	30−49	11.297	5.018	0.098	0.070	16.483
	50−64	9.595	4.236	0.077	0.058	13.966
	>65	7.093	3.124	0.055	0.042	10.315
	Average	9.099	4.029	0.076	0.055	13.260

^1^ Total EDI of lignans (mg/person/day) = EDI of lignans from sesame seeds (mg/person/day) + EDI of lignans from sesame oil (mg/person/day).

**Table 8:** The average contributive composition percentage (%) of sesame seed and oil for individual lignans.

Gender	Type	Sesamin	Sesamolin	Sesamol	Sesaminol	Total
Males	Sesame seeds	14.7	6.1	0.0	0.1	21.0
	Sesame oil	53.8	24.3	0.6	0.4	79.0
	Sum	68.5	30.4	0.6	0.4	100.0
Female	Sesame seeds	17.6	7.3	0.0	0.1	25.0
	Sesame oil	51.1	23.1	0.5	0.3	75.0
	Sum	68.6	30.4	0.6	0.4	100.0
Average	Sesame seeds	16.1	6.7	0.0	0.1	23.0
	Sesame oil	52.4	23.7	0.6	0.3	77.0
	Sum	68.6	30.4	0.6	0.4	100.0

## References

[1] Wan Y., Li H., Fu G., Chen X., Chen F., Xie M. (2015). The relationship of antioxidant components and antioxidant activity of sesame seed oil. J. Sci. Food Agric..

[2] Sarkis J.R., Michel I., Tessaro I.C., Marczak L.D.F. (2014). Optimization of phenolics extraction from sesame seed cake. Sep. Purif. Technol..

[3] Korea Customs Service, Korea Trade Statistics Import/Export by Commodity. http://www.customs.go.kr.

[4] Durazzo A., Lucarini M., Camilli E., Marconi S., Gabrielli P., Lisciani S., Gambelli L., Aguzzi A., Novellino E., Santini A. (2018). Dietary Lignans: Definition, Description and Research Trends in Databases Development. Molecules.

[5] Pathak N., Bhaduri A., Rai A.K., Mérillon J.M., Ramawat K. (2017). Sesame: Bioactive compounds and health benefits. Bioactive Molecules in Food.

[6] Rangkadilok N., Pholphana N., Mahidol C., Wongyai W., Saengsooksree K., Nookabkaew S., Satayavivad J. (2010). Variation of sesamin, sesamolin and tocopherols in sesame (*Sesamum indicum* L.) seeds and oil products in Thailand. Food Chem..

[7] Mahendra Kumar C., Singh S.A. (2015). Bioactive lignans from sesame (*Sesamum indicum* L.): Evaluation of their antioxidant and antibacterial effects for food applications. J. Food Sci. Technol..

[8] Wu M.S., Aquino L.B.B., Barbaza M.Y.U., Hsieh C.L., De Castro-Cruz K.A., Yang L.L., Tsai P.W. (2019). Anti-Inflammatory and Anticancer Properties of Bioactive Compounds from *Sesamum indicum* L.—A Review. Molecules.

[9] Majdalawieh A.F., Mansour Z.R. (2019). Sesamol, a major lignan in sesame seeds (*Sesamum indicum*): Anti-cancer properties and mechanisms of action. Eur. J. Pharmacol..

[10] Lee J., Choe E. (2006). Extraction of Lignan Compounds from Roasted Sesame Oil and their Effects on the Autoxidation of Methyl Linoleate. J. Food Sci..

[11] Suja K.P., Jayalekshmy A., Arumughan C. (2005). Antioxidant activity of sesame cake extract. Food Chem..

[12] Lee J., Lee Y., Choe E. (2008). Effects of sesamol, sesamin, and sesamolin extracted from roasted sesame oil on the thermal oxidation of methyl linoleate. LWT Food Sci. Technol..

[13] Dar A.A., Verma N.K., Arumugam N. (2015). An updated method for isolation, purification and characterization of clinically important antioxidant lignans—Sesamin and sesamolin, from sesame oil. Ind. Crops Prod..

[14] Michailidis D., Angelis A., Aligiannis N.I., Mitakou S., Skaltsounis L.A. (2019). Recovery of sesamin, sesamolin and minor lignans from sesame oil using solid support free liquid-liquid Extraction and Chromatography techniques and evaluation of their enzymatic inhibition properties. Front. Pharmacol..

[15] Moazzami A.A., Kamal-Eldin A. (2006). Sesame seed is a rich source of dietary lignans. J. Am. Oil Chem. Soc..

[16] Bhatnagar A.S., Hemavathy J., Gopala Krishna A.G. (2015). Development of a rapid method for determination of lignans content in sesame oil. J. Food Sci. Technol..

[17] Dar A.A., Arumugam N. (2013). Lignans of sesame: Purification methods, biological activities and biosynthesis—A review. Bioorganic Chem..

[18] Jeon H., Kim I.-H., Lee C., Choi H.-D., Kim B.H., Akoh C.C. (2013). Discrimination of Origin of Sesame Oils Using Fatty Acid and Lignan Profiles in Combination with Canonical Discriminant Analysis. J. Am. Oil Chem. Soc..

[19] Wang L., Zhang Y., Li P., Wang X., Zhang W., Wei W., Zhang X. (2012). HPLC Analysis of Seed Sesamin and Sesamolin Variation in a Sesame Germplasm Collection in China. J. Am. Oil Chem. Soc..

[20] Hemalatha S. (2004). Ghafoorunissa Lignans and tocopherols in Indian sesame cultivars. J. Am. Oil Chem. Soc..

[21] Moazzami A.A., Andersson R.E., Kamal-Eldin A. (2006). HPLC Analysis of Sesaminol Glucosides in Sesame Seeds. J. Agric. Food Chem..

[22] Kim J.H., Seo W.D., Lee S.K., Lee Y.B., Park C.H., Ryu H.W., Lee J.H. (2014). Comparative assessment of compositional components, antioxidant effects, and lignan extractions from Korean white and black sesame (*Sesamum indicum* L.) seeds for different crop years. J. Funct. Foods.

[23] Kim K.S., Park S.H., Choung M.G. (2006). Nondestructive Determination of Lignans and Lignan Glycosides in Sesame Seeds by Near Infrared Reflectance Spectroscopy. J. Agric. Food Chem..

[24] Liu Z., Saarinen N.M., Thompson L.U. (2006). Sesamin is one of the major precursors of mammalian lignans in sesame seed (*Sesamum indicum*) as observed in vitro and in rats. J. Nutr..

[25] Kamal-Eldin A., Appelqvist L.Å., Yousif G. (1994). Lignan analysis in seed oils from four Sesamum species: Comparison of different chromatographic methods. J. Am. Oil Chem. Soc..

[26] Shyu Y.-S., Hwang L.S. (2002). Antioxidative activity of the crude extract of lignan glycosides from unroasted Burma black sesame meal. Food Res. Int..

[27] KNHANE (2017). Korea National Health and Nutrition Examination Survey.

[28] FDA (2001). Guidance for Industry: Bioanalytical Method Validation.

[29] Ajit G., Uma D., Manonmani S., Vinothkumar B., Rajesh S. (2019). Diversity Analysis of Sesame Lignans in 40 Sesame Collections in Tamil Nadu, India. Int. J. Curr. Microbiol. App. Sci..

